# Guanfacine for Treatment of Anxiety and Panic-Induced Vasovagal Syncope in the Intensive Care Unit

**DOI:** 10.7759/cureus.86075

**Published:** 2025-06-15

**Authors:** Shixie Jiang, Diego Nolasco, Richard Czuma, Brandon Janssen, Matthew Gunther

**Affiliations:** 1 Psychiatry, University of Florida College of Medicine, Gainesville, USA; 2 Psychiatry, Edward Hines Jr. Veterans Administration Medical Center, Hines, USA; 3 Pulmonary, Critical Care and Sleep Medicine, University of Florida College of Medicine, Gainesville, USA; 4 Psychiatry and Behavioral Sciences, Stanford University School of Medicine, Palo Alto, USA; 5 Psychiatry, University of Southern California, Los Angeles, USA

**Keywords:** anxiety, critical care, guanfacine, intensive care unit, panic, syncope

## Abstract

This case report describes the successful use of oral guanfacine, a centrally acting alpha-2 agonist, for treating panic-induced vasovagal syncope in a 68-year-old intensive care unit (ICU) patient recovering from lung transplant and cardiac complications. The patient experienced recurrent episodes of severe anxiety and syncope triggered by physical therapy, unresponsive to standard anxiolytics. Guanfacine was chosen given its less pronounced cardiovascular effects and modulation of norepinephrine, and titrated over 10 days, leading to complete resolution of symptoms without adverse effects or recurrence. The patient resumed rehabilitation and guanfacine was successfully tapered prior to discharge. This case highlights guanfacine as a potentially useful anxiolytic option for ICU patients even with cardiovascular vulnerability as it exhibits less pronounced cardiovascular effects and may therefore be considered safer than other alpha-2 agonists.

## Introduction

Anxiety can occur in an estimated 45% of patients in the intensive care unit (ICU) setting and leads to greater severity of critical illness and prolonged lengths of stay [1]. It can provoke prominent physiologic responses in addition to typical psychological distress, particularly in those with underlying cardiac or respiratory exacerbations [2]. Standard non-pharmacological and pharmacological interventions for anxiety disorders often require extended treatment durations to achieve chronic relief. This prolonged timeline often renders them less ideal for acutely ill patients who need immediate symptomatic management. Furthermore, acute pharmacologic options often involve the use of benzodiazepines and other anxiolytics (e.g., hydroxyzine, quetiapine) which can incur various deleterious effects in an ICU population due to their anticholinergic properties [3]. Dexmedetomidine is a common intravenous alpha-2 agonist that has been widely used in the ICU setting, often for sedation, pain, and agitation management [4]. It has preliminarily demonstrated some efficacy for anxiety, but alpha-2 agonists as a whole have yet to be thoroughly studied for treatment of acute anxiety in the hospitalized setting [4]. Moreover, dexmedetomidine in particular commonly causes bradycardia and hypotension, and its use is restricted to the ICU setting. Guanfacine is an oral alpha-2 agonist that is Food and Drug Administration (FDA) approved for attention-deficit/hyperactivity disorder (ADHD). Its dosing range varies from 0.5 to 4 mg [5]. It possesses a much higher selectivity for alpha-2 receptors and less preference for imidazoline receptors [6,7]. Thus, it may be less likely to cause cardiovascular adverse effects while still providing anxiolysis. To our knowledge, we present the first case examining the use of guanfacine to manage marked physiologic anxiety and panic-induced vasovagal syncope.

## Case presentation

The patient was a 68-year-old male with no previous psychiatric diagnoses and a medical history relevant for coronary artery disease status-post stent placement, myocardial infarction, and fibrotic lung disease admitted to the ICU for a bilateral lung transplant. The day after his lung transplant, he developed sudden cardiac arrest in the form of pulseless ventricular tachycardia, with a heart catheterization revealing a new left bundle branch block and left ventricular dysfunction. He underwent emergent coronary artery bypass grafting and was additionally placed on veno-arterial extracorporeal membrane oxygenation. Our critical care psychiatry consultation team was consulted three weeks later after recovery for recurrent anxiety preventing progression of his ICU care. His primary team had attempted a regimen of mirtazapine, olanzapine, and hydroxyzine with no success.

On evaluation, the patient reported intermittent episodes of worry accompanied by difficulty breathing, restlessness, muscle tension, palpitations, and diaphoresis. He denied any significant mood or trauma symptoms. Many aspects of his medical care caused episodes of anxiety as they reminded him of his medical complications while in the ICU. Most saliently, any attempted physical or occupational therapy (PT/OT) interventions led to panic attacks and subsequent vasovagal syncope. For example, if asked to stand with the PT/OT team, he would immediately develop a strong fear of dying followed by hyperventilation, palpitations, brief tachycardia, and syncope. He was evaluated by cardiology several times and his work-up only demonstrated a mild asymptomatic bradycardia (average heart rate 55 beats per minute). Our team diagnosed him with an other specified anxiety disorder with a Hospital Anxiety and Depression Scale (HADS) score of 19. We recommended discontinuation of his other psychotropics and initiation of guanfacine 0.5 mg in the morning and 1 mg at night for his anxiety and sleep disturbances. His symptoms rapidly improved throughout the next week, with titration of guanfacine to 1 mg twice a day. By day 10, he no longer experienced panic attacks or episodes of vasovagal syncope with PT/OT interventions and reported a HADS score of 5. Guanfacine was continued for the remainder of his hospitalization with no recurrence of symptoms or worsening of bradycardia, and successfully tapered leading up to hospital discharge.

## Discussion

Neurochemical theories suggest dysregulated functioning of norepinephrine plays a significant role in the etiology of anxiety and panic symptoms. Perceived stress leads to the release of corticotropin-releasing hormone and subsequent modulation of norepinephrine projections throughout the locus coeruleus, amygdala, insula, and medial prefrontal cortex [8]. This can often present clinically as prominent physiological symptoms (e.g., palpitations, shortness of breath, tachycardia, diaphoresis) compared to psychological or ruminative anxiety [9]. In vasovagal syncope, an emotional and/or physical trigger (e.g., PT/OT and stress associated with intervention) initiates a reflex leading to activation of vagal afferents mediated by norepinephrine. This leads to an increased parasympathetic response, especially at the sinus and atrioventricular nodes, leading to a subsequent decrease in heart rate. Concurrently, there is a withdrawal of sympathetic activity leading to decreased arterial and venule tone. This vasodilation leads to a decrease in total peripheral resistance and venous return. The amalgamation of the decrease in heart rate and vasodilation leads to a decrease in cardiac output, blood pressure, and cerebral blood flow, thus causing a temporary loss of consciousness [10].

Based on the putative role of norepinephrine in anxiety and vasovagal syncope pathophysiology, we proposed to empirically trial guanfacine given its potent alpha-2 agonist properties. It notably has higher alpha-2 selectivity than other alpha-2 agonists [6,7], thus making it potentially ideal for treatment of this particular ICU patient due to the core physiologic characteristics of his anxiety in addition to safety considerations given his extensive cardiac history and active bradycardia. Other common adverse effects (e.g., dizziness, sedation, and dry mouth) were monitored for daily and the risks of these occurring were considered less concerning given the hypothesized benefits. Higher alpha-2 selectivity leads to less preferential affinity for the imidazoline receptor, thus causing less bradycardia and hypotension [6]. Furthermore, its lack of anticholinergic properties compared to other common anxiolytics creates a unique role for its use in the ICU due to the high risk of delirium. Though for any patients already delirious, preliminary data suggests that guanfacine may still serve a role for symptomatic relief in this population [11,12]. Finally, clinicians may find the long half-life of guanfacine (17 hours) to be potentially beneficial for ease of daily administration and the tapering process with extended use. 

## Conclusions

This case illustrates the potential utility of guanfacine as a safe and helpful pharmacologic option for physiologic anxiety and panic-related vasovagal syncope in critically ill patients, particularly when standard anxiolytics are poorly tolerated or ineffective. In ICU settings, where patients often have complex cardiac and respiratory comorbidities, pharmacologic interventions must be chosen carefully to avoid exacerbating underlying conditions. Guanfacine’s unique mechanism of action, minimal anticholinergic burden, and relatively favorable cardiovascular profile make it a compelling candidate for use in medically vulnerable populations. In this case, its introduction led to rapid resolution of panic symptoms and syncope, allowing the patient to engage in rehabilitation and continue recovery without further psychiatric or cardiovascular complications. While this is a single case, the observed benefit supports further exploration of oral alpha-2 agonists as viable options for acute anxiety management in the ICU, especially for patients in whom sympathetic hyperarousal contributes significantly to symptom burden.
